# Three-Dimensional-Printed Polymeric Cores for Methane Hydrate Enhanced Growth

**DOI:** 10.3390/polym15102312

**Published:** 2023-05-15

**Authors:** Andrey Stoporev, Rail Kadyrov, Tatyana Adamova, Evgeny Statsenko, Thanh Hung Nguyen, Murtazali Yarakhmedov, Anton Semenov, Andrey Manakov

**Affiliations:** 1 Department of Petroleum Engineering, Kazan Federal University, Kremlevskaya Street 18, 420008 Kazan, Russia; rail7777@gmail.com (R.K.); e.statsenko@yahoo.com (E.S.); thanhtu154@gmail.com (T.H.N.); manakov@niic.nsc.ru (A.M.); 2 Nikolaev Institute of Inorganic Chemistry SB RAS, Lavrentieva Avenue 3, 630090 Novosibirsk, Russia; adamova@niic.nsc.ru; 3 Department of Physical and Colloid Chemistry, Gubkin University, Leninsky Prospekt 65, Building 1, 119991 Moscow, Russiasemyonovanton@mail.ru (A.S.)

**Keywords:** gas hydrates, methane, 3D printing, hydrate growth, polymeric core

## Abstract

Polymeric models of the core prepared with a Raise3D Pro2 3D printer were employed for methane hydrate formation. Polylactic acid (PLA), acrylonitrile butadiene styrene (ABS), carbon fiber reinforced polyamide-6 (UltraX), thermoplastic polyurethane (PolyFlex), and polycarbonate (ePC) were used for printing. Each plastic core was rescanned using X-ray tomography to identify the effective porosity volumes. It was revealed that the polymer type matters in enhancing methane hydrate formation. All polymer cores except PolyFlex promoted the hydrate growth (up to complete water-to-hydrate conversion with PLA core). At the same time, changing the filling degree of the porous volume with water from partial to complete decreased the efficiency of hydrate growth by two times. Nevertheless, the polymer type variation allowed three main features: (1) managing the hydrate growth direction via water or gas preferential transfer through the effective porosity; (2) the blowing of hydrate crystals into the volume of water; and (3) the growth of hydrate arrays from the steel walls of the cell towards the polymer core due to defects in the hydrate crust, providing an additional contact between water and gas. These features are probably controlled by the hydrophobicity of the pore surface. The proper filament selection allows the hydrate formation mode to be set for specific process requirements.

## 1. Introduction

Gas hydrates are ice-like solids composed of water and gas molecules. The water molecules form defined cage structures and networks via hydrogen bonding, which are stabilized by the enclathration of the gas molecules [1]. Gas hydrates have wide distribution in nature [2], with large gas storage capacity (about 160/140 volumes of methane/carbon dioxide per hydrate volume); this encourages researchers to employ them as gas storage containers and gas/water absorbers [3,4]. In addition, the different hydrate-forming ability of a components mixture allows gases to be separated via hydrate phase enrichment with hydrate-forming components [5,6]. Hydrate-based water desalination is also possible [7]. However, the efficiency of hydrate-based solutions turned out to be inferior to traditional methods, primarily due to the high energy consumption for gas compression and reactor cooling as well as the low hydrate formation rate. The latter can be increased, for example, by adding water-soluble surfactants and helping hydrate formers (dioxolane, tetrahydrofuran, cyclopentane, etc.) [8,9] or by intensive mixing [10]. The stirring also helps resolve the slow nucleation issue. In turn, cold energy from liquefied natural gas reduces the cost of maintaining the required temperature for the hydrate formation process. These options undoubtedly increase the hydrate formation’s energy efficiency and make it competitive with well-developed techniques [11,12,13,14]. However, such solutions can decrease the hydrate gas capacity, need promoter regeneration/water remediation, and significantly complicate the apparatus for continuously producing hydrates, especially at high pressures. A possible alternative is the creation of efficient surfaces catalyzing the nucleation and growth of hydrates, which would ensure the formation of hydrate particles immediately after water–gas contact under suitable thermodynamic conditions with a satisfactory hydrate growth rate. Hydrate nucleation most often occurs near the liquid–gas interface due to the necessary supersaturation of the fluid with dissolved gas in this region [15]. The influence of artificially introduced surfaces in contact with water and gas, which serve as centers of heterogeneous nucleation, on the hydrate’s formation is intensively studied [16]. So far, the characteristics of surfaces that favor the gas hydrates formation are not yet sufficiently understood. One can only indicate the importance of the particle surface’s composition and morphology on the hydrate nucleation’s kinetics. The development of such substrates requires preliminary scientific research. However, it is reliably known that an increase in the water–gas contact surface also makes it possible to accelerate hydrate nucleation even at low driving forces. Moreover, the water-to-hydrate conversion will be more significant in the case of an increased water–gas contact surface at the same aqueous phase volume [17]. Indeed, after water surface overgrowth, the further hydrate growth rate is controlled by the diffusion of gas and/or water molecules into the reaction zone. Thus, porous materials containing uniformly distributed water are promising systems for obtaining hydrates [18,19]. Moreover, studying the gas hydrates formation and decomposition in natural porous media is relevant for understanding the formation process of these compounds in nature and the production of gas from hydrate-bearing sediments [20,21,22]. Utilizing various visualization methods allows the features of the processes under consideration to be identified [23,24], making it possible to develop ways to control them in the future.

At the same time, not only surfactants promote hydrate phase growth [25,26] but also the chemical modification of surfaces [27,28,29]. Moreover, the growth efficiency of hydrates of various gases (or structures) in the porous medium in the presence of surfactants varies significantly [30]. An intensive upward hydrate growth was recently found for poplar wood shavings [31]. Unfortunately, the cited work does not provide a complete analysis of the shavings’ surface and the presence of possible contaminants in the shavings (e.g., contaminant chain lubricants and mineral oils). Anyway, it does show that the presence of certain surfaces can stimulate intense upward hydrate growth.

Various materials of natural origin (e.g., sand and clay) can be considered the most studied porous ones for accelerating hydrate formation. Other promising porous materials for the production of hydrates are organic and metal–organic frameworks [32,33], carbon materials [34,35], cellulose [19,36,37], and powdered cryogels [17]. It should be noted that the kinetics of hydrate growth in such systems is often improved due to the increased water–gas interface and some influence of the surface structure on nucleation (although such studies are few). Also of note is the potential of porous media design for hydrate obtaining [35]. Three-dimensional printing of various materials is actively developing and allows for optimizing production processes [38,39,40,41,42,43,44,45]. In the case of gas hydrates, such an approach makes it possible to vary the medium’s parameters for obtaining hydrate in a wide range, allowing optimizing artificial materials for developing a particular hydrate-based technology.

The current work employs porous materials in an unusual role, namely to control the direction of hydrate growth (while maintaining a high growth rate). It is proposed to use 3D-printed plastic cores as the porous media.

## 2. Materials and Methods

Pure methane (99.99 vol%; Moscow Gas Refinery, Moscow Region, Russia) and deionized water were used as the hydrate-forming substances. Polylactic acid (PLA), acrylonitrile butadiene styrene (ABS), and polycarbonate (ePC) filaments were purchased from eSUN (Shenzhen, China); polyamide-6 filled with 27 mass% of short carbon fibers (UltraX, Guangzhou, China) and thermoplastic polyurethane (PolyFlex, Baltic, OH, USA) were also used. The pore space of the Berea sandstone was taken as a model for polymeric cores.

**Figure 1:**
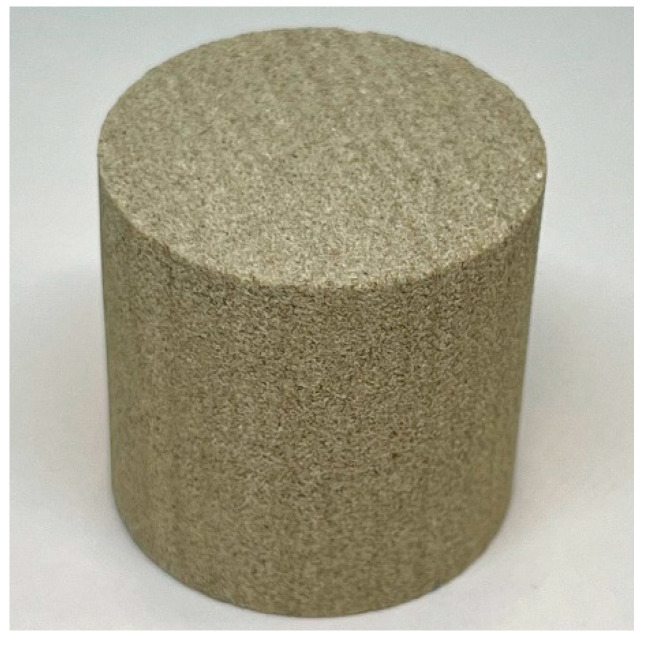
A core of the Berea sandstone (“Split rock” type) used for the research.

Berea sandstone is a type of sedimentary rock that has been widely used for various laboratory studies and pore-scale digital modeling. Its high porosity and permeability make it an ideal material for studying fluid flow in porous media (for example, see [46,47,48,49]). It is widely used to study the effects of different types of fluids on rock properties [50,51,52]. By understanding fluids’ interactions with porous media, the behavior of reservoirs can be predicted with high accuracy. In this research, a core sample related to the “Split rock” variation of the sandstone from the Berea Formation in Bedford, OH, USA, with a porosity of 19.6% and a water permeability of 81.22 mD, was used (Figure 1).

### 2.1. X-ray Computed Tomography

A micro- and nanofocusing X-ray computed tomography system (General Electric V|tome|X S 240; Wunstorf, Germany) was used. Two samples of Berea sandstone cut to 4 × 4 × 4 mm and 2 × 2 × 2 mm were selected to create 3D model of the core. The samples were scanned using a microfocus tube at a voltage of 100 kV and a current of 100 mA. The scanning resolution was 3.4 and 5.8 µm, respectively. Then, the 3D models were reconstructed in the Phoenix dato|x software. Segmentation and processing of samples were carried out in the Avizo software. Sandstone models with dimensions 4 × 4 × 4 mm and 2 × 2 × 2 mm (in the latter case, only PLA* sample) were enlarged 9 and 20 times, respectively. A rectangular parallelepiped with dimensions of 27 × 15 × 7 mm cut out of the obtained models was used for 3D printing. The pore space was segmented using the Magic Wand function, and the effective porosity along the Z scale was selected using the Axis connectivity function. Finally, a mesh model (STL format) based on effective porosity subtracted from the parallelepiped volume was obtained using the Meshing operation.

The 3D-printed polymeric cores were rescanned at 100 kV, 130 mA (the resolution of all samples was 32 μm). The pore space was segmented for each obtained digital model. Effective porosity along Z-axis and equivalent diameters distribution over the volume were obtained.

### 2.2. Three-Dimensional Printing

**Figure 2:**
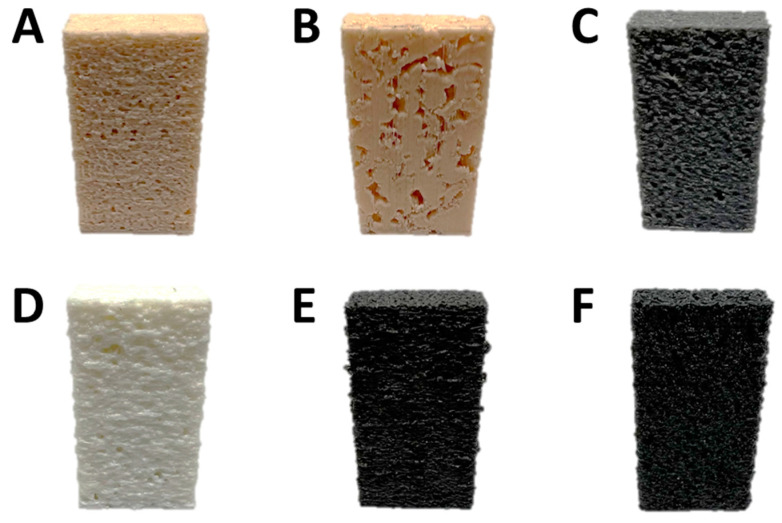
Three-dimensional-printed plastic cores: (**A**)—PLA, (**B**)—PLA* (400 µm extrusion width), (**C**)—ABS, (**D**)—PolyFlex, (**E**)—UltraX, (**F**)—ePC.

A Raise3D Pro2 3D printer (USA) was employed. The first model (extracted from the 4 × 4 × 4 mm sample) was printed with 5 different filaments (PLA, ABS, UltraX, PolyFlex, and ePC), and the second one (2 × 2 × 2 mm sandstone) was printed using PLA plastic. The printing parameters are shown in Table 1. The exterior of the obtained 3D-printed polymeric samples is shown in Figure 2.

### 2.3. Gas Hydrate Formation Test

**Figure 3:**
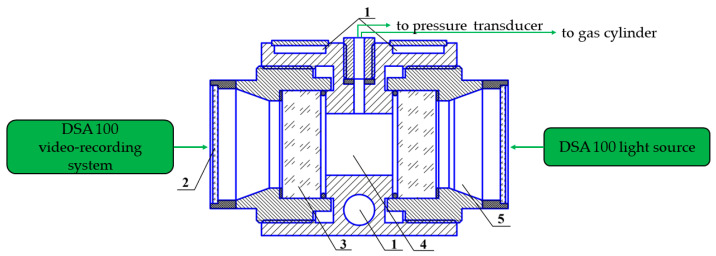
High-pressure visual cell; 1—coolant circulating channels connected to a thermostat, 2—shielding windows, 3—sapphire windows, 4—cell’s working volume, 5—air space with a moisture absorber.

A 27.5 mL in-volume visual cell with two leucosapphire windows (Figure 3) coupled with the Drop Shape Analyzer DSA 100 (Kruss, Hamburg, Germany) was employed. Methane hydrate formation was studied at 1 °C and about 9.1 MPa (subcooling was 11 °C). The morphology of the hydrate being formed was registered with the DSA 100 photo and video-recording system.

When the growth of the hydrate stopped for 1–2 h (no visual and pressure changes), the system was usually kept under the specified conditions for about 12 h; then, the pressure was fixed, and the hydrate was decomposed. A 700G30 reference pressure transducer (Fluke, Everett, WA, USA) with a measuring range of 0–34.5 MPa and a maximal error of 0.017 MPa was used to check the initial and final pressures in the cell (without recording). A temperature sensor (Pt100) was embedded in the cell body to control temperature constancy. Methane consumption was calculated based on *P*, *T* data using the Peng−Robinson equation. X-ray computed tomography data on polymer inserts volume were used when calculating the free cell space. Pressure and volume deviations may result in some errors in the counted amount of gas passed into the hydrate.

## 3. Results

### 3.1. Three-Dimensional-Printed Cores X-ray Computed Tomography Characterization

After 3D printing, each plastic model was rescanned at 32 µm resolution. After the reconstruction of 3D models, the pore space was segmented for each obtained digital model. After that, the volumes of effective porosity were identified along the *Z* axis, for which the distribution of equivalent diameters over the volume was obtained (see Table 2 and Figures S1 and S2). Figure S2 shows orthogonal tomographic sections in the XZ and YZ planes of the studied samples, 3D visualization of the pieces, and void space.

To describe the structure of effective porosity in more detail, an effective pore diameter distribution depending on their volumes relative to the volume of all pores was also plotted (Figure S1). In this case, individual pores of effective porosity are distinguished based on the algorithm for dividing pore chambers according to the pore throats.

There is a slight relative deviation of the porosity coefficient in most printed core models within 0.5%. The most different sample PolyFlex has a porosity ratio of 16.3% with only 7.0% of effective porosity. Due to the features and materials printing, the distribution of effective pores can differ greatly from the original digital model.

### 3.2. Methane Hydrate Growth with 3D-Printed Cores

**Figure 4:**
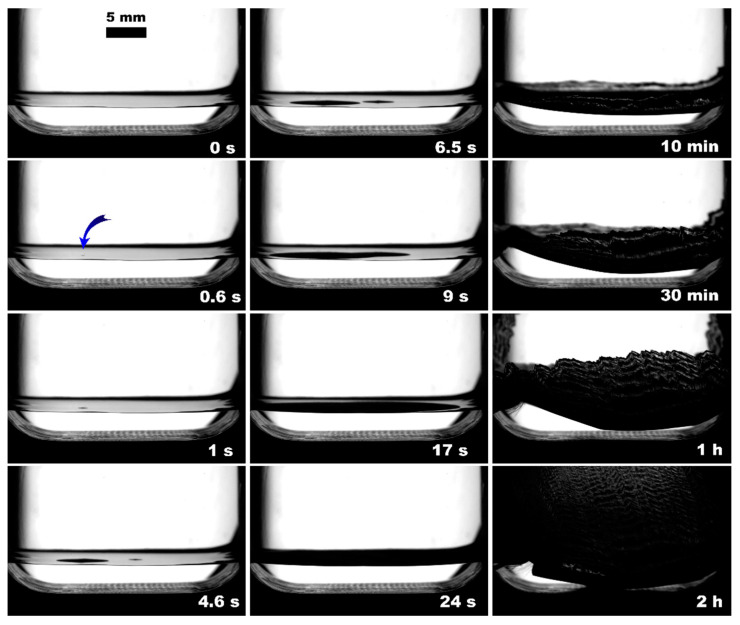
Methane hydrate formation from 3 mL of deionized water without insertion; blue arrow indicates the hydrate onset site.

**Figure 5:**
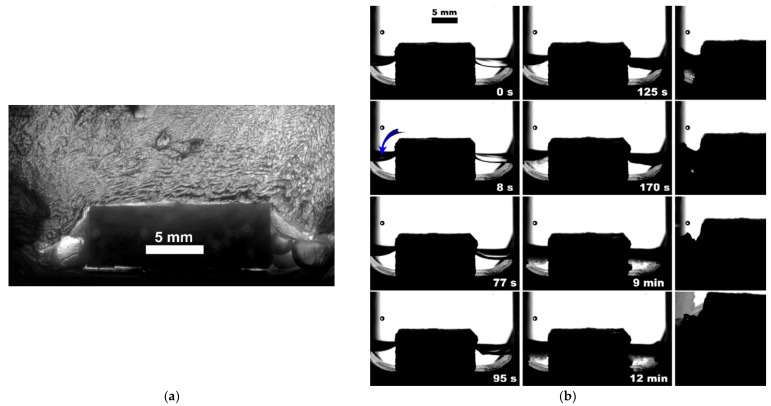
Methane hydrate formation from 3 mL of deionized water with PLA* after 12 h since hydrate onset (**a**) and 2 mL of deionized water with PLA (**b**) insertions; blue arrow indicates the hydrate onset site.

**Figure 6:**
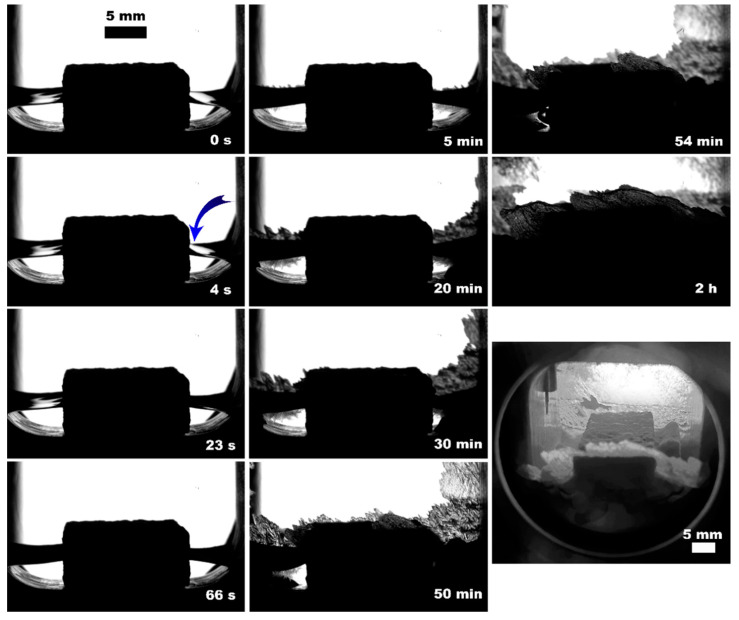
Methane hydrate formation from 2 mL of deionized water with PolyFlex insertion; the blue arrow indicates the hydrate onset site; the lower right photo shows the system’s appearance after 12 h since the hydrate onset.

**Figure 7:**
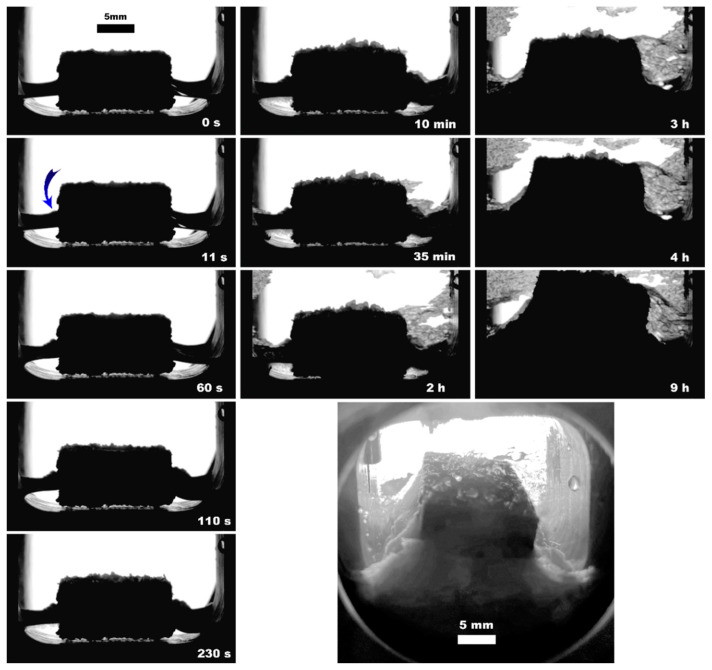
Methane hydrate formation from 2 mL of deionized water with UltraX insertion; the blue arrow indicates the hydrate onset site; the lower right photo shows the system’s appearance after 12 h since the hydrate onset.

**Figure 8:**
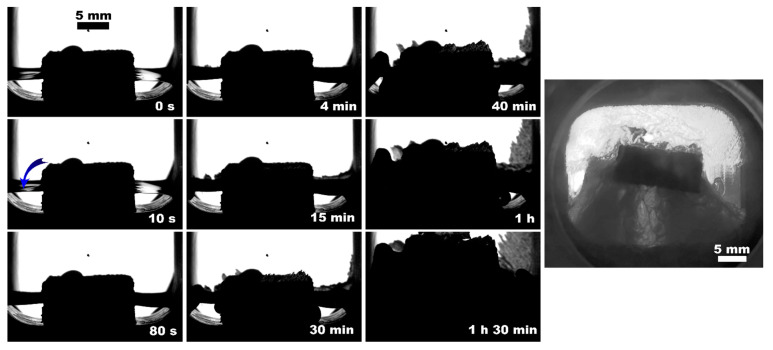
Methane hydrate formation from 2 mL of deionized water with ePC insertion; the blue arrow indicates the hydrate onset site; the right photo shows the system’s appearance after 12 h since the hydrate onset.

**Figure 9:**
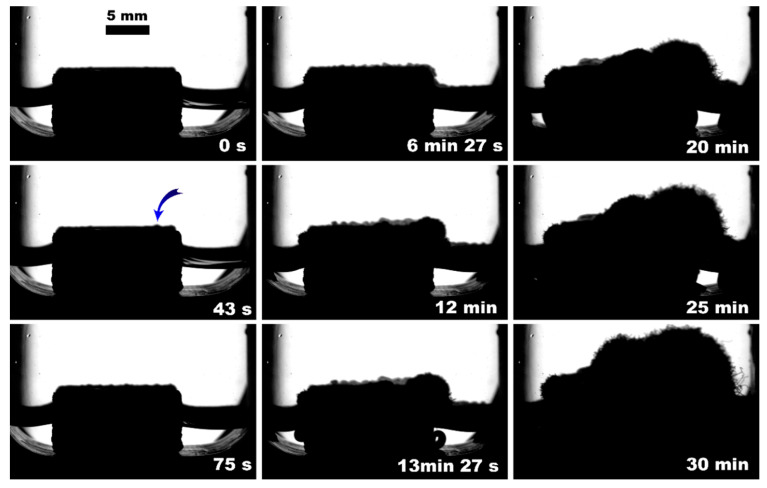
Methane hydrate formation from 2 mL of deionized water with ABS insertion; the blue arrow indicates the hydrate onset site.

The observed nucleation events and further methane hydrate growth parameters are summarized in Table 3 and Figure 4, Figure 5, Figure 6, Figure 7, Figure 8 and Figure 9. It should be noted right away that a detailed study of nucleation was not carried out at this stage. Only the sites of the hydrate growth onset were identified in a few cases. In the presented photographs (Figure 4, Figure 5, Figure 6, Figure 7, Figure 8 and Figure 9) and videos (see Supplementary; Videos S1–S9), the time is counted almost from when the hydrate crystallization center appears.

## 4. Discussion

As noted above, this work did not study nucleation. At the same time, it should be stressed that nucleation in such systems can occur at the water–gas, water–gas–metal, water–gas–sapphire (for example, see [53]), and water–gas–polymer interfaces with the gas-in-water concentration playing a vital role in the nucleation process [54]. The literature has repeatedly noted that hydrate nucleation at contact with a polymer is infrequent (for instance, on fluoroplastic and polycarbonate [55,56,57]). In most instances, nucleation undoubtfully prevailed at the water–gas or water–gas–metal interface (see Figure 4, Figure 5, Figure 6, Figure 7, Figure 8 and Figure 9, Figures S3 and S5, and Videos S1–S9).

**Figure 10:**
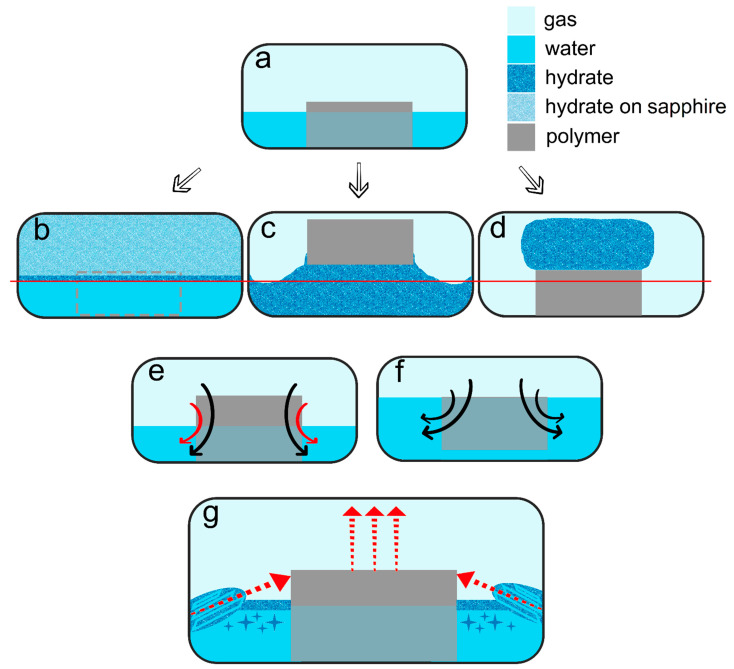
(**a**) The initial view of a system; (**b**) hydrate formation with PLA*, PolyFlex, and without a polymeric core; (**c**) hydrate formation with PLA, UltraX, ePC, and ABS (5 mL of water); (**d**) hydrate formation with ABS (2 mL of water); the red row line in subfigures (**b**–**d**) corresponds to the initial water level; (**e**) gas transfer through both upper and side surfaces for partial filling of porous space; (**f**) gas transfer through upper surface for complete filling of porous space; (**g**) growth of massive hydrate bodies from the cell’s walls (for PLA, ePC, UltraX) and blowing of hydrate crystals into the volume of water (for PLA, ePC, UltraX, and ABS); side arrows indicate the direction of hydrate bodies growth; the vertical ones show the motion of the core.

As for the hydrate growth, Figure 10 summarizes the findings. This work revealed that the porous polymer insertions placed within the water volume could enhance methane hydrate formation (up to complete water-to-hydrate conversion for PLA sample compared to 4–5% for the water–gas system with no insertion; Table 3) with the type of polymer affecting the hydrate formation mode. All polymer cores except PolyFlex and PLA* promoted the hydrate growth. In the case of PolyFlex and PLA*, hydrate growth was observed only on the surfaces of water–gas and sapphire windows (Figure 10b). The formation of massive hydrate bodies both in the water volume and on the polymer cores’ surface was not registered. Since the effective porosity differs slightly for PLA and PLA* samples (19.4 and 18.8%, Table 2), but the extrusion width differed by a factor of 2 (200 versus 400 µm), it can be assumed that the main reason for the difference in the water-to-hydrate conversion for these samples (about 100 and 6%, Table 3) is the trajectory of connected pores (does not allow transfer between water and gas for PLA*). Large pores (6 and 8% of pores with a diameter of 2.8 and 4 mm; Figure S1) may also be an affecting factor. In the case of PolyFlex, the low value of effective porosity seems to hinder the transfer between water and gas. However, the polymer itself may also have some influence (for example, the adhesion energy of the hydrate to the surface of a given polyurethane).

When it comes to the water volume effect, this is not an influencing factor if the polymer core does not affect the hydrate growth (compare the water-to-hydrate conversion for different water volumes with no insertion and with PLA*). The hydrate forms first on the water–gas surface and then slowly grows on the sapphire window’s surface. If the samples sink in water, growth should proceed similarly to a system without a core. Models made from ABS and ePC do not sink in water, even under methane pressure (Figure 10f). At the same time, with an increase in the filling of the porous volume with water from partial (with 2 mL of water) to complete (with 5 mL of water), the efficiency of hydrate growth decreases by two times (see Table 3). This suggests that the upper surface of the core and its ends make approximately the same contribution to the transfer of reagents between the aqueous and gas phases during hydrate growth. Indeed, the surface reduction due to water volume increase for ABS and ePC equals 56 and 59% (Table 3), with the water-to-hydrate conversion decreasing by 49–50%.

**Figure 11:**
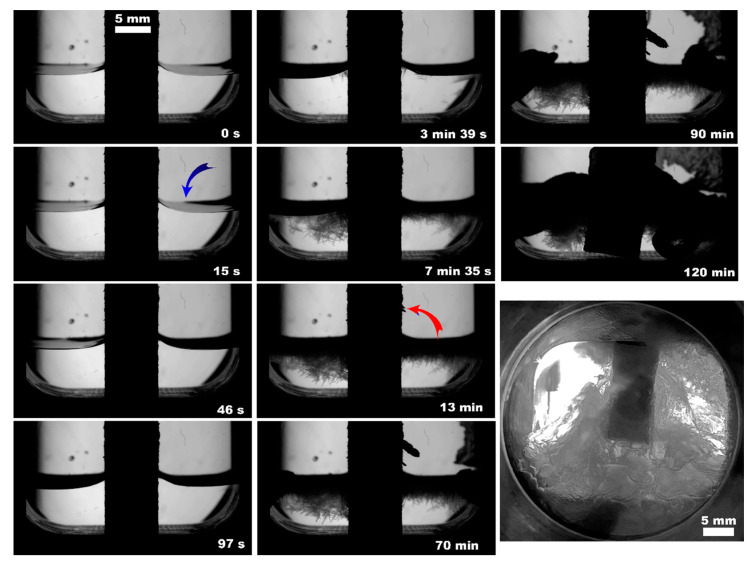
Methane hydrate formation from 5 mL of deionized water with PLA insertion; the blue arrow indicates the front of initial hydrate growth, the red one shows the start of hydrate growth at the sapphire; the lower right photo shows the system’s appearance after 12 h since the hydrate onset.

On the other hand, three-phase water–gas–polymer contact (partial filling of porous space) was provided in experiments with PLA, regardless of the water volume (due to the different core positions; Figure 5b and Figure 11, Videos S2 and S7). For 5 mL of water, the surface of the polymer insert in the gas phase even increased by 21%. At the same time, the conversion for 2 and 5 mL of water differed by more than 2 times (100% and 43%, respectively). This may indicate the anisotropy of the direction of the connected channels in the printed PLA core. Conducting studies with polymer bodies with straight channels at different angles to the water surface might reveal the optimal configuration. Another reason for this behavior may be the driving force of hydrate growth. Table 3 shows that the final pressure in the system varies slightly for different volumes of water.

From the point of view of the growth regimen and hydrate morphology, the presence of a polymeric core contributes to the manifestation of three main features. (1) The type of polymer allows hydrate growth to be directed either under the insert (transfer of gas into the water volume) or on its top (migration of water into the gas volume; compare Figure 10c,d); compare Figure 5b, Figure 7, Figure 8, Figure 11, Figures S4 and S5 with Figure 9 (also Videos S2 and S5–S7 with S3). The ABS core (in the case of 2 mL of water; partial filling of the pore volume) ensures the growth of the hydrate due to the transfer of water to gas. This exciting feature can be used to separate water from non-hydrate-forming water-soluble impurities. At the same time, this feature was not observed in experiments with 5 mL of water (complete filling of the pore space). This again indicates the importance of the experiment’s configuration and the possible anisotropy in the coupled channel network. In addition, repeated hydrate formation just after hydrate dissociation revealed growth predominantly proceeded in the bulk of water (Video S4). This indicates that some channels responsible for the upward water migration remained inactive after the hydrate decomposition (probably due to the formation of water–gas layers). (2) A blowing of hydrate crystals into the volume of water (Figure 10g; for PLA—Video S2 and S7, Figure 11 (7.5 min); for ePC—Videos S6 and S9; for UltraX—Video S5; for ABS—Videos S6 and Figure S4 (picture 5)). After such blowing, the hydrate often grew in bubbles from the ends of the insert into the water volume (apparently, after the hydrate film overgrew the outlets of the channels). (3) Sometime after blowing out the hydrate crystals, hydrate arrays start to grow from the steel cell’s walls in the direction of the polymer core (Figure 10g). Notably, this growth mode does not manifest itself without a polymer core. Obviously, the polymer cannot “attract” the hydrate growing from the wall. It seems to be likely that the morphology of the hydrate crust formed on the water surface is responsible for this. It can be assumed that defects are created in it, providing additional contact between water and gas.

The observed features of hydrate growth are probably controlled by the hydrophobicity of the pore surface. It is evident that the hydrate film growth rate along the gas−water interface inside the pores depends on the thermobaric conditions, the efficiency of heat dissipation, the type of gas, but weakly on the pore size (for sizes greater than 100 nm), and the gas flow rate (for example, [58]). However, the latter parameters can significantly impact the hydrate’s ability to linger in these pores or grow continuously through them. In our work, hydrate growth through pores was observed in a static mode (without gas flow) and depended on the type of polymer (due to different water wettability and hydrate adhesion) and channel size/effective porosity (compare data for PLA, PLA*, and PolyFlex samples). Pressure and temperature will also significantly affect the growth regimen and its efficiency. The influence of these parameters is planned to be studied in future works. These developments will be helpful in the design of polymer membranes on the surface of the water for its hydrate-based desalination/purification. Directional hydrate growth can also benefit hydrate-based gas storage and separation technologies. The proper filament selection will allow the hydrate formation mode to be set for specific process requirements.

## 5. Conclusions

Different types of polymeric cores and variations of pore sizes/effective porosity (including an anisotropy in the coupled channels network) were revealed to be affecting the methane hydrate growth. The presented approach makes it possible to control the direction of hydrate growth as well as to achieve the complete water-to-hydrate conversion (for PLA core) by properly selecting the polymer. The direction of the connected pores seems to influence the transition considerably. It is assumed that varying the type of material, its structure (the shape and size of the channels), and the functionalization of the surface of these channels will make it possible to achieve separation of the hydrate and residual solution directly during the hydrate growth. Thus, studying the formation of hydrates in a porous medium can become the basis for developing gas hydrate technology for water purification. Achieving a high rate of hydrate production on such membranes will also allow them to be considered for storing and transporting gases (methane, hydrogen, natural gas), and carbon dioxide capture.

## Figures and Tables

**Table 1:** Printing options.

Polymer	Extrusion Width (µm)	Layer Thickness (µm)	Extrusion Temperature (°C)	Table Temperature (°C)
PLA	200	50	205	60
PLA*	400	200	205	60
ABS	200	50	250	100
PolyFlex	200	50	225	60
UltraX	200	50	300	100
ePC	200	50	260	100

**Table 2:** Characteristics of the printed samples.

Sample	Porosity (%)	Effective Porosity (%)	Effective Porosity Volume (mm^3^)	Total Model Volume (mm^3^)	Equivalent Pore Diameter (mm)
Digital model	19.6	19.6	556.1	2835.0	1.2 ± 0.7
PLA	20.9	19.4	497.6	2569.7	1.0 ± 0.6
PLA*	20.4	18.8	426.5	2262.1	2.0 ± 1.2
ABS	20.4	18.4	500.2	2717.9	1.0 ± 0.5
PolyFlex	16.3	7.0	167.0	2385.9	1.3 ± 0.6
UltraX	20.4	17.1	429.9	2515.9	1.0 ± 0.5
ePC	20.0	18.5	455.4	2464.5	1.4 ± 0.8

**Table 3:** List of experiment parameters.

Sample	Water Volume (mL)	*T* (°C)	Initial *P* (MPa)	Final *P* (MPa)	Water-to-Hydrate Conversion (%)	Δ*S* (%) ^1^
No insertion	3	1	9.20	9.14	4.1	–
	5	1	9.02	8.89	4.9	–
PLA	2	1	8.72	7.63	100.6 ^2^	–
	5	1	8.98	7.65	42.7	21
PLA*	3	1	9.25	9.16	5.6	–
	2	1	9.25	9.19	5.9	–
ABS	2	1	9.20	8.51	65.2	–
	2 (r1) ^3^	1	9.10	8.45	61.3	–
	5	1	8.99	8.04	30.7	−56
	5 (r1)	1	8.88	8.52	11.8 ^5^	−56
	5 (r2) ^4^	1	8.88	7.95	29.9	−56
PolyFlex	2	1	9.25	9.17	7.2	–
UltraX	2	1	9.28	8.73	52.9	−60
ePC	2	1	9.17	8.55	59.3	–
	5	1	9.18	8.31	28.8	−59

^1^ Changing the surface of the polymer insert in the gas phase while switched from the partial to complete filling of porous space (upper and about half of the side surfaces for 2 mL of water, and only upper surface for 5 mL of water); in the case of PLA, the surface increased due to the different core position; ^2^ Estimated volume of polymer inserts and pressure measurement accuracy can cause an error in water-to-hydrate conversion; ^3^ r1—re-obtaining the hydrate after hydrate dissociation near equilibrium conditions (at 14–15 °C); r2—re-obtaining the hydrate after complete hydrate dissociation at 35 °C for 2 h; ^4^ r2—re-obtaining the hydrate after complete hydrate dissociation at 35 °C for 2 h; ^5^ This run lasted just about 40 min.

## Data Availability

The data presented in this study are available on request from the corresponding author.

## Supplementary Materials

The following supporting information can be downloaded at: https://www.mdpi.com/article/10.3390/polym15102312/s1, effective porosity data (pore diameters distribution (Figure S1) and structure of 3D-printed samples (Figure S2)); additional hydrate morphology photos (Figures S3–S5); Videos S1–S9: formation of methane hydrate growth at 1 °C and about 9.1 MPa from deionized water with varying 3D-printed polymer cores.
