# Supplementary material for: Three-Dimensional-Printed Polymeric Cores for Methane Hydrate Enhanced Growth
Source: Polymers (Basel). 2023 May 15;15(10):2312. doi: 10.3390/polym15102312 (PMC10221765; doi:10.3390/polym15102312)
Supplement: Supplementary file 1 [file polymers-15-02312-s001.zip › polymers-2351414-supplementary.pdf]

Supplementary Material

Three-dimensional-Printed Polymeric Cores for Methane Hydrate Enhanced Growth

Andrey Stoporev [1,*], Rail Kadyrov [1], Tatyana Adamova [2], Evgeny Statsenko [1], Thanh Hung Nguyen [1], Murtazali Yarakhmedov [3], Anton Semenov [3] and Andrey Manakov [1,2]

[1] Department of Petroleum Engineering, Kazan Federal University, Kremlevskaya Street 18, 420008 Kazan, Russia; rail7777@gmail.com (R.K.); e.statsenko@yahoo.com (E.S.); thanhtu154@gmail.com (T.H.N.); manakov@niic.nsc.ru (A.M.)

[2] Nikolaev Institute of Inorganic Chemistry SB RAS, Lavrentieva Avenue 3, 630090 Novosibirsk, Russia

[3] Department of Physical and Colloid Chemistry, Gubkin University, Leninsky Prospekt 65, Building 1, 119991 Moscow, Russia; semyonovanton@mail.ru (A.S.)

* Correspondence: andrey.stoporev@kpfu.ru or stopor89@bk.ru

Contents

Effective porosity data S3

Methane hydrate morphology S12

Effective porosity data

Pore diameters distribution

Volume fraction of pores with a certain equivalent diameter was calculated based on the total volume of effective porosity.

Figure S1. Pore diameters distribution depending on their volumes relative to the volume of effective porosity; the samples names are specified on the graphs.

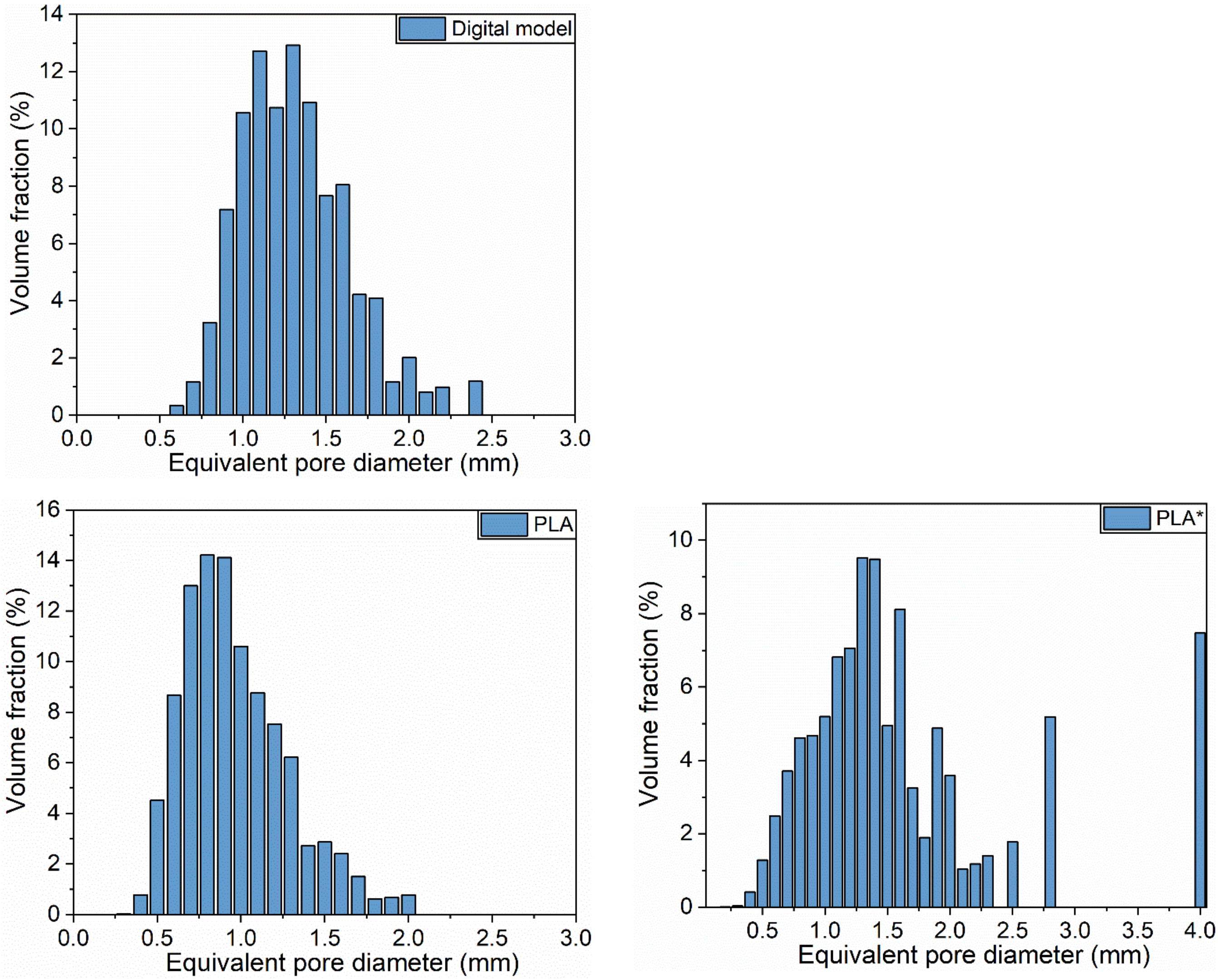

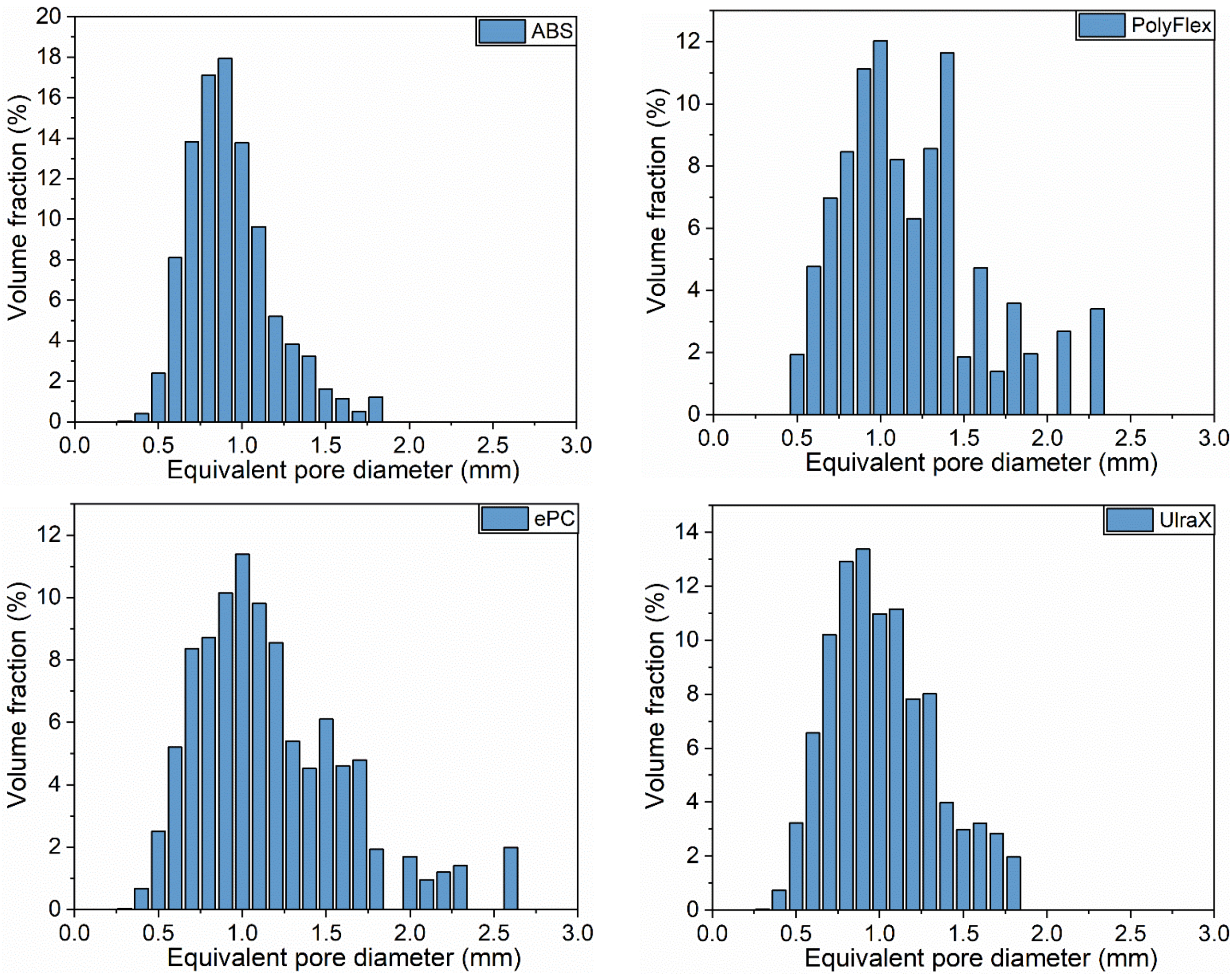
ABS
PolyFlex
ePC
UlraX
Volume fraction (%)
Equivalent pore diameter (mm)
0.0
0.5
1.0
1.5
2.0
2.5
3.0
0
2
4
6
8
10
12
14
16
18
20

The structure of 3D-printed samples

Digital model (for 200 μm extrusion width)

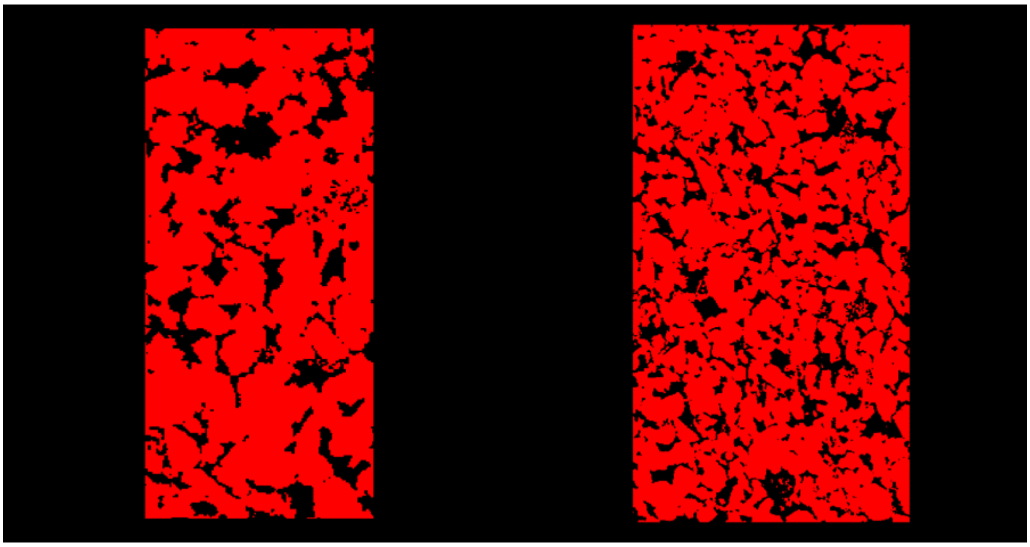

XZ YZ

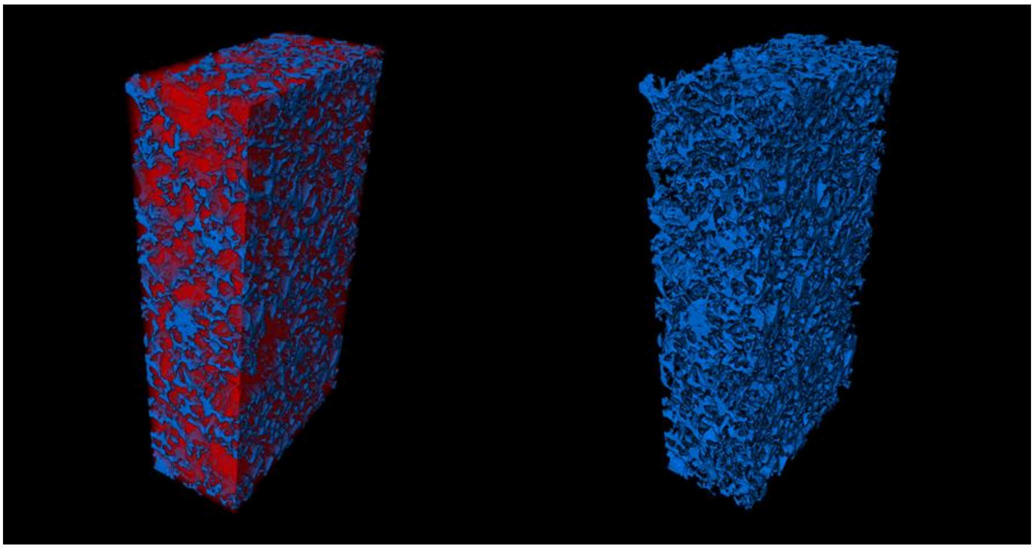

3D visualization of the sample 3D visualization of void space

PLA

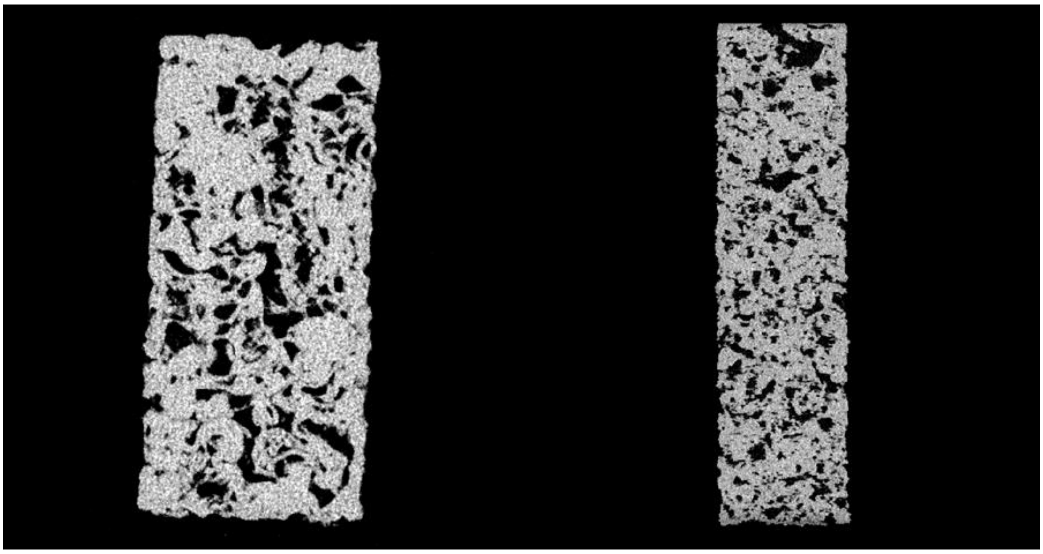

XZ YZ

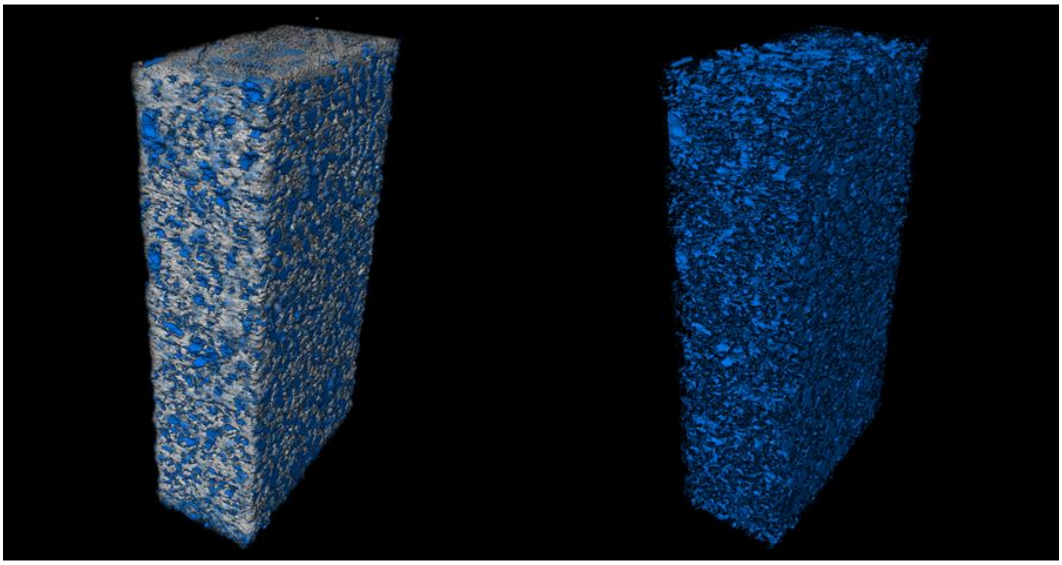

3D visualization of the sample 3D visualization of void space

PLA*

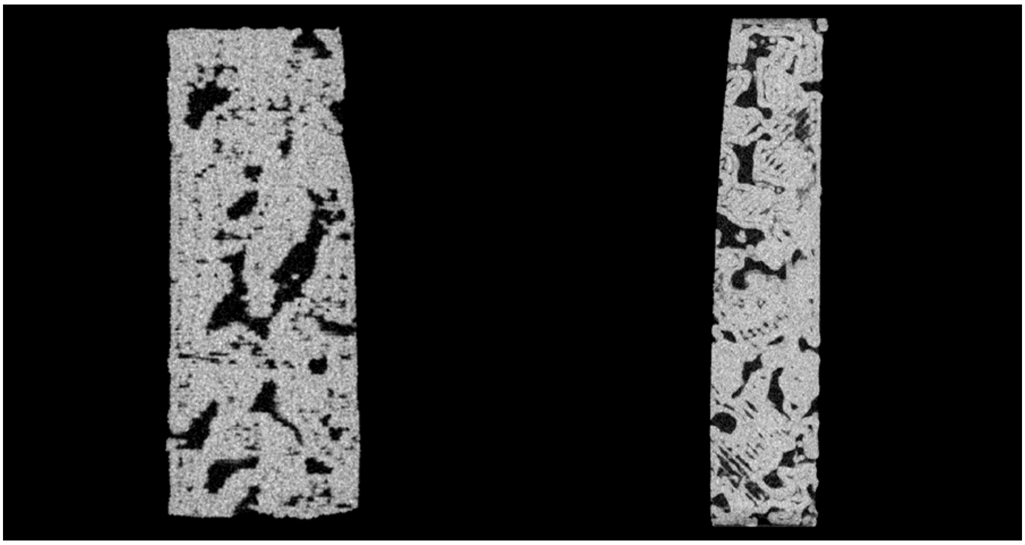

XZ YZ

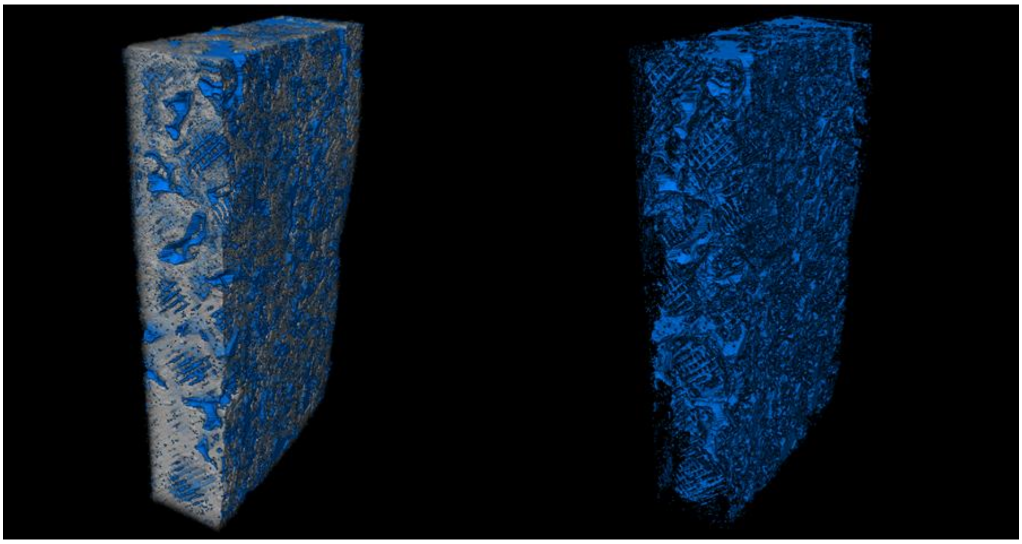

3D visualization of the sample 3D visualization of void space

PolyFlex

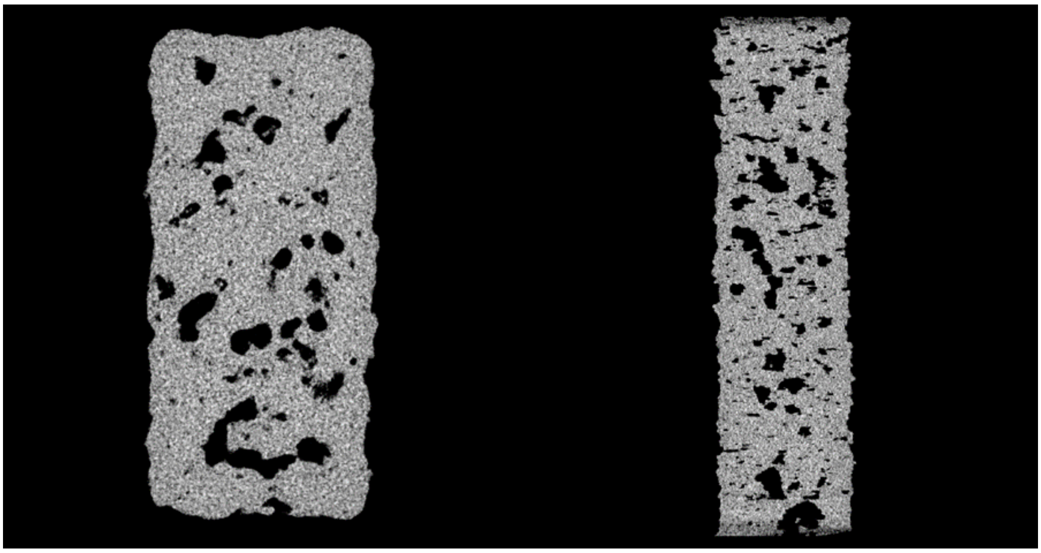

XZ YZ

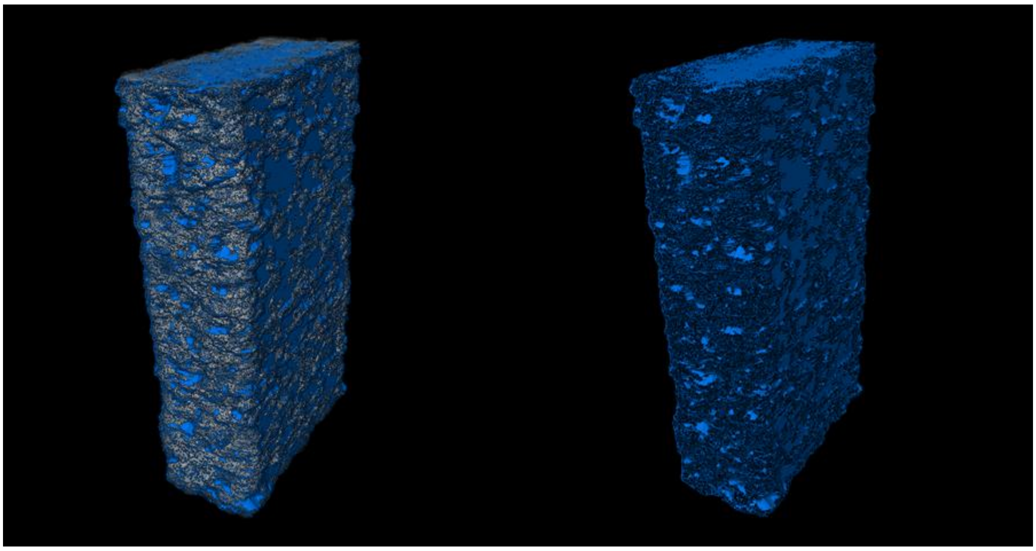

3D visualization of the sample 3D visualization of void space

UltraX

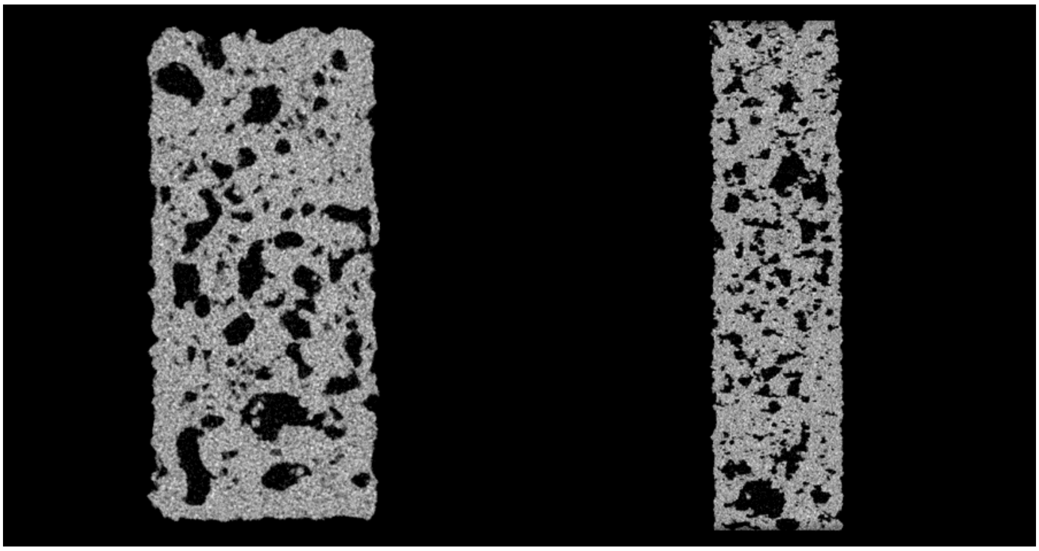

XZ YZ

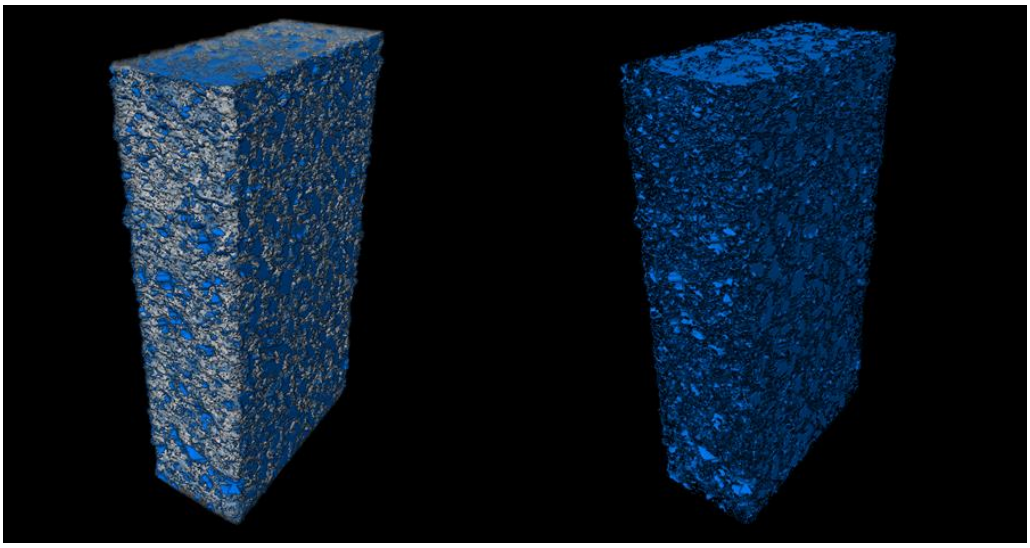

3D visualization of the sample 3D visualization of void space

ABS

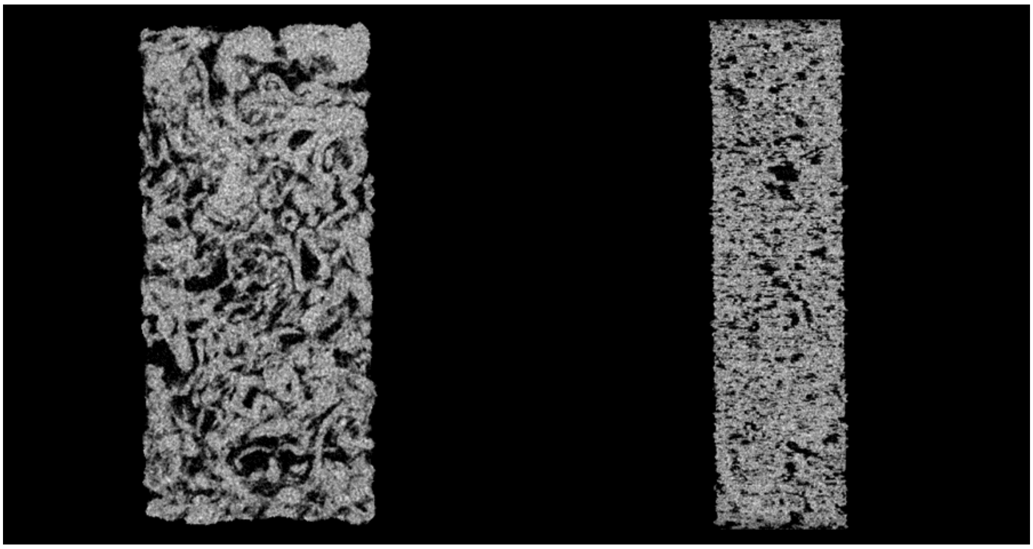

XZ YZ

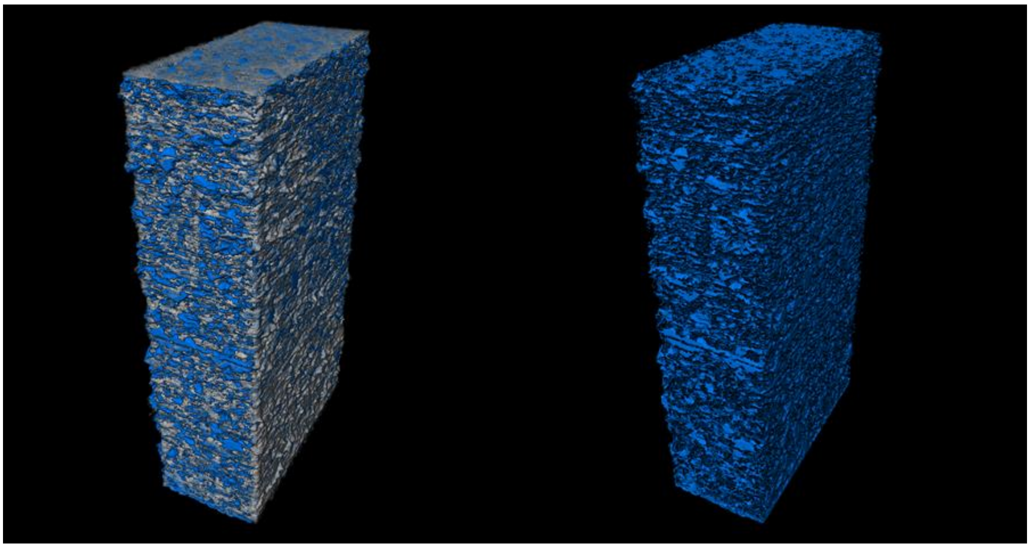

3D visualization of the sample 3D visualization of void space

ePC

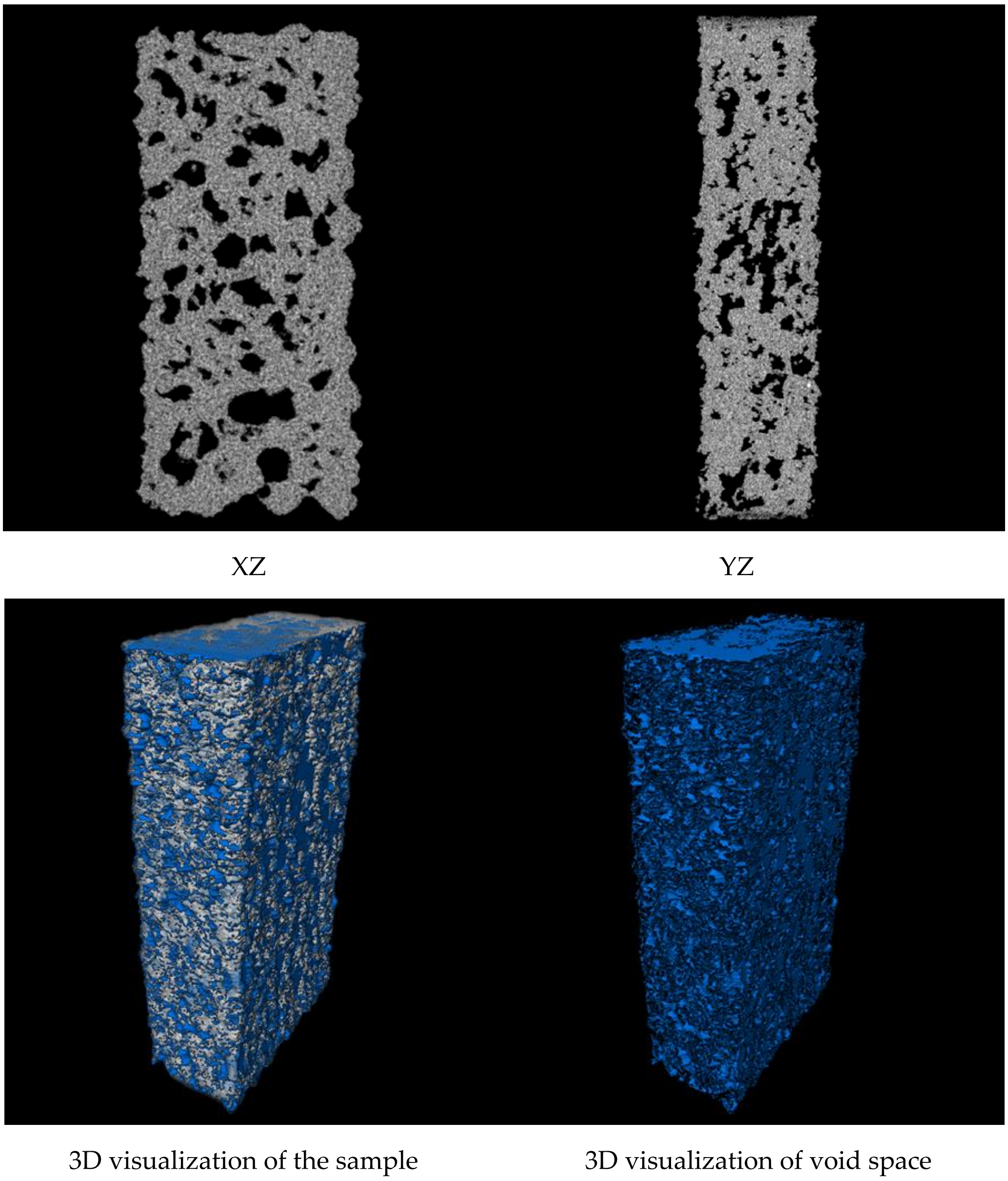

Figure S2. XZ and YZ orthogonal tomographic sections of the studied samples, their 3D visualization, and the void space; the rectangular parallelepiped dimensions are 27x15x7 mm.

Hydrate morphology

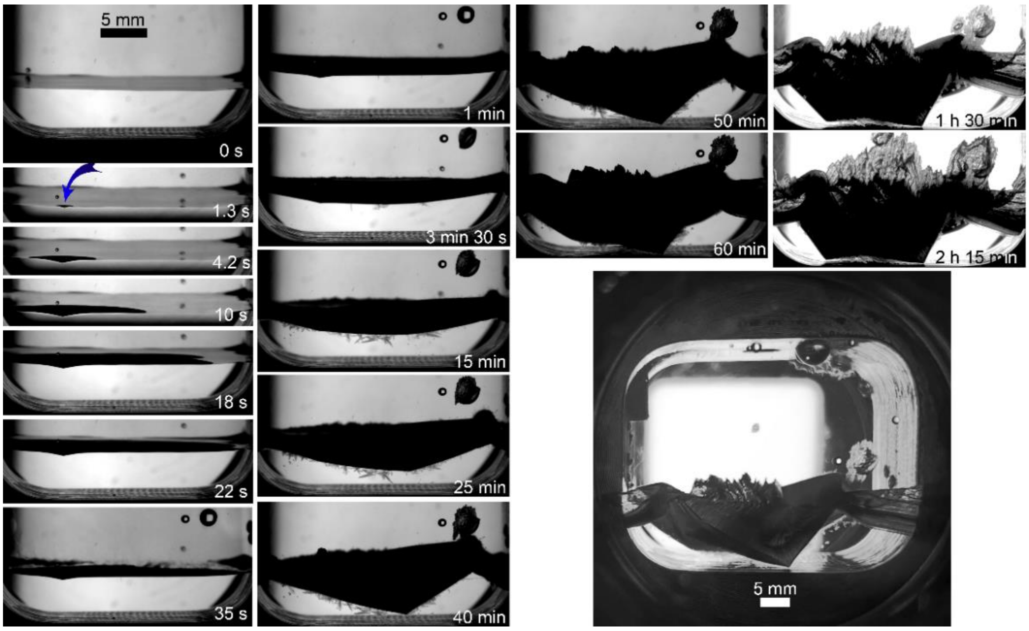

Figure S3. Methane hydrate formation from 5 ml of deionized water without an insertion; blue arrow indicates the hydrate onset site; the lower right photo shows the system's appearance after 12 h since the hydrate onset.

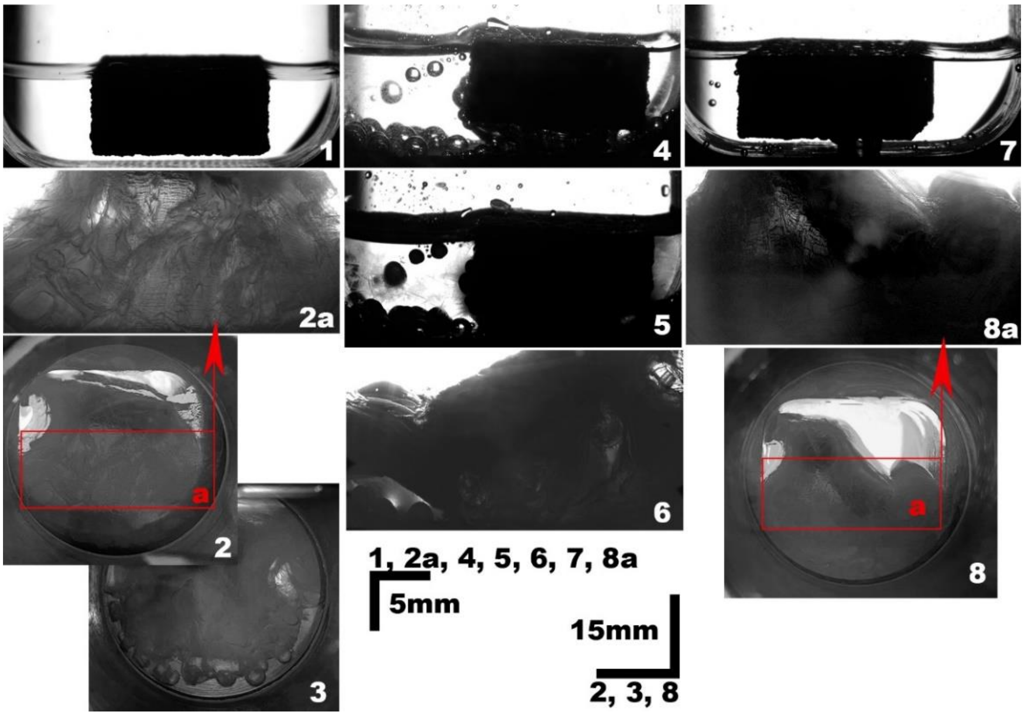

Figure S4. Methane hydrate formation from 5 ml of deionized water with ABS insertion; 1-3 – 1st hydrate formation, 4-6 – re-obtaining the hydrate (for 40 min) after hydrate dissociation near equilibrium conditions (at 14-15°C); 7-8 – re-obtaining the hydrate after complete hydrate dissociation at 35° for 2 h; picture 5 shows hydrate crystals blown into the water volume.

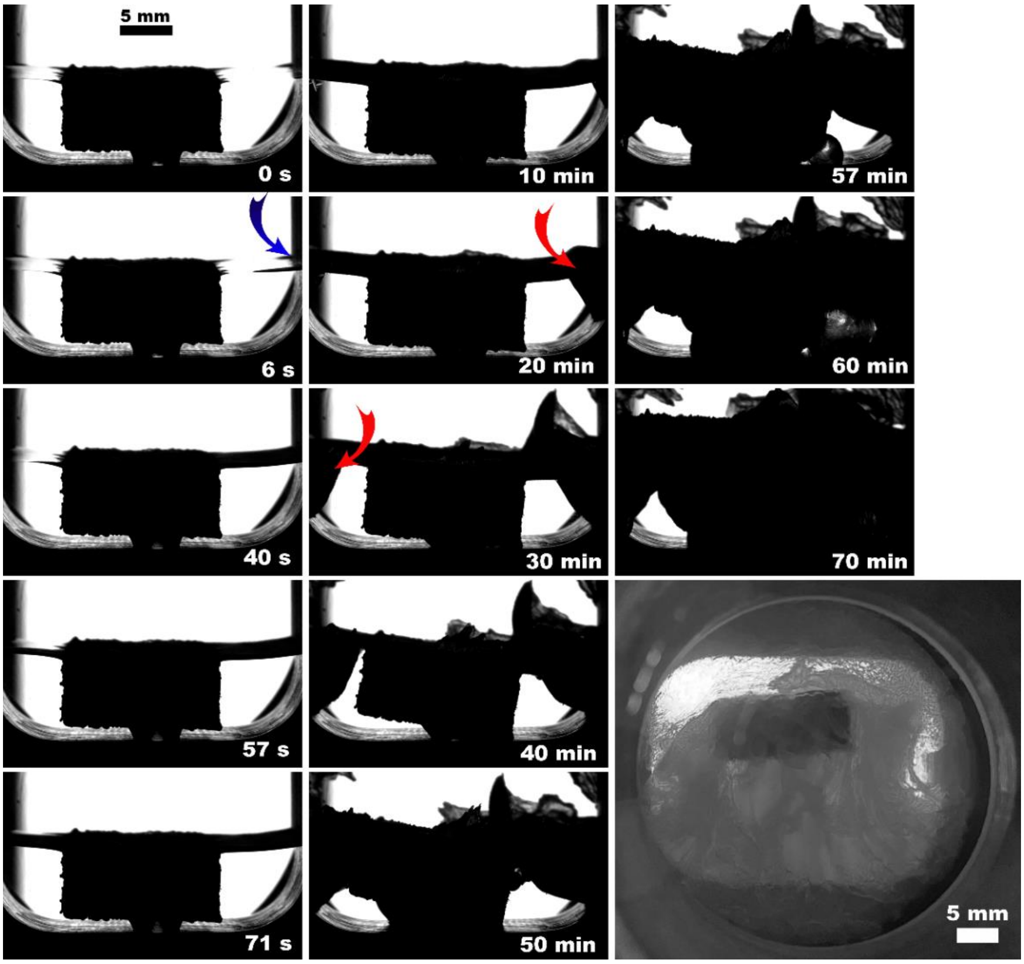

Figure S5. Methane hydrate formation from 5 ml of deionized water with ePC insertion; blue arrow indicates the hydrate onset; the red ones show hydrate arrays growing from cell walls; the lower right photo shows the system's appearance after 12 h since the hydrate onset.
